# A triple-band terahertz metamaterial perfect absorber for biomedical applications and biomarker detection

**DOI:** 10.1371/journal.pone.0342575

**Published:** 2026-07-06

**Authors:** Mohammad Mahmudul Alam Mia, Md. Ruhul Amin, Sayed Shifat Ahmed, Md. Eyakub Ali, Jannatul Nayem Novera

**Affiliations:** Electrical and Electronic Engineering, RTM Al-Kabir Technical University, Sylhet, Bangladesh; Purdue University, UNITED STATES OF AMERICA

## Abstract

This study presents a triple-band perfect metamaterial absorber with a compact microstructured design, achieving near-unity absorption rates of 99.92%, 99.97%, and 99.58% at three distinct terahertz resonance frequencies of 0.925 THz, 1.71 THz, and 2.7675 THz, respectively, for advanced biomedical biosensing applications. The work represents a theoretical and numerical proof-of-concept investigation based on full-wave electromagnetic simulations and refractive-index-assisted biosensing analysis, demonstrating the feasibility of high-sensitivity terahertz detection of biological analytes. The proposed absorber consists of concentric copper ring resonators combined with a central 7-shaped snowflake pattern, developed on an FR-4 dielectric substrate and backed by a continuous copper ground plane. Numerical simulations using the FDTD method were combined with a genetic algorithm approach, enabling the design of high-performance terahertz metamaterials, yielding three sharp, high-Q resonances with near-perfect absorption and ultra-narrow linewidths characteristics. The proposed sensor also demonstrates excellent capabilities including breast cancer cell identification with peak sensitivity of 10,142.86 GHz/RIU, infectious agent recognition, and polarization insensitivity over 0–90°, alongside enhanced absorption tolerance that supports clinical deployment. To evaluate biosensing capability, refractive-index-assisted numerical modeling was performed using literature-reported dielectric properties of biological analytes, including cancer cells, viruses, glucose concentrations, blood components, intracellular materials, and biological tissues. The proposed sensor achieves a maximum sensitivity of 529 GHz/RIU at the third resonance mode with strong linear resonance shifts and high spectral selectivity. Owing to its multi-band terahertz sensor facilitates and strong confinement of electric and magnetic fields at the resonant frequencies enhances the device’s sensitivity to minimal changes in the surrounding medium, allowing for precise, label-free detection of biological analytes including viral variants, malaria pathogens, glucose concentrations, hemoglobin elements, classification of diabetes severity and can also differentiate in anemia categorization across diverse biomedical applications.

## 1. Introduction

In recent years, the development of highly sensitive terahertz (THz) devices has emerged as a pivotal research frontier, driven by their transformative potential in biomedical diagnostics, therapeutic monitoring, and early disease detection. The global health landscape continues to be profoundly challenged by infectious diseases, cancer, and chronic metabolic disorders, necessitating the development of versatile and high-throughput diagnostic technologies. According to the World Health Organization’s World Health Statistics 2023 report, infectious diseases remain among the leading causes of death worldwide, with tuberculosis alone affecting an estimated 10.8 million people in 2023 [1]. Cancer accounts for nearly 10 million deaths annually, making it a critical target for early detection and monitoring. Additionally, metabolic disorders such as diabetes affect over 537 million adults globally, with numbers projected to rise significantly in the coming decade [2,3]. Malaria continues to impose a heavy burden, with millions of cases reported annually across endemic regions [4]. Despite ongoing control efforts, the global malaria treatment market is projected to grow from USD 1.89 billion in 2025 to USD 3.11 billion by 2035, reflecting a compound annual growth rate (CAGR) of 5.1% [5]. Given the increasing global burden of diseases such as cancer which remains the second leading cause of mortality worldwide according to the World Health Organization, the need for rapid, reliable, and non-invasive diagnostic solutions is more urgent than ever. Terahertz plasmonic metasurface absorbers (PMAs) enable compact, scalable biosensing platforms. Metamaterial-photonics synergy advances next-generation sensors meeting rigorous healthcare requirements for early screening and monitoring [6]. The increasing incidence of biomolecular diseases necessitates advanced diagnostic platforms capable of rapid, sensitive, and non-invasive detection. THz radiation (0.1–10 THz) combines low photon energy with sensitivity to molecular vibrations, enabling non-ionizing, label-free interrogation of biological materials. This makes THz technology ideal for probing biomarkers linked to cancer, viral infections, diabetes, malaria, and neurological disorders. Perfect Metamaterial Absorbers (PMAs), engineered periodic structures exhibiting near-unity absorption at resonant frequencies, enhance light–matter interactions at subwavelength scales, facilitating selective and sensitive detection of dielectric changes [7]. Narrowband PMAs, characterized by narrow spectral linewidths and high-quality factors, are particularly suited for precise refractive index sensing, resolving subtle spectral shifts caused by trace biomolecular binding. This synergy supports the development of next-generation biosensors critical for global health monitoring and disease control. Metamaterials with tunable subwavelength features enable perfect absorption and high refractive index (RI) sensitivity [8]. Multi-band metamaterial absorbers offer versatile, simultaneous detection of multiple biomarkers across distinct frequencies, making them ideal for real-time monitoring of diverse bioanalytes including glucose and cancer cells.

Despite significant progress, the design and optimization of multiband THz metamaterial sensors remain challenging and prone to fabrication challenges due to intricate geometry and narrow bandwidth requirements. P. Upender *et al.* in paper [9], developed a graphene-based metamaterial biosensor with four SRRs and a central graphene ring, achieving 1.354 THz absorption, tunability via chemical potential, and high sensing performance (S = 1.7 THz/RIU, FoM = 165.09, Q = 112.5), suitable for virus and cancer cell detection. While the design shows promise for terahertz sensing and optoelectronic devices, its practical implementation is limited by the complexity of multilayer fabrication and the sensitivity of graphene’s performance to chemical potential tuning. Comparatively, In [10], a tunable metamaterial absorber operating in the terahertz range was developed by B. Khodadadi *et al.* using a four-layer structure (graphene/SiO_2_/Si/Au), demonstrating strong polarization insensitivity and high absorption peaks of 98.32% and 98.49% at 4.3 THz and 7.35 THz, respectively. While its suitability for biomedical sensing applications such as cancer detection and glucose monitoring is notable, practical deployment may be hindered by its dependency on complex material stacking and limited Q-factor tunability. Moreover Y. Cao *et al.* was proposed a reflective terahertz metasurface microfluidic sensor in [11], utilizing a self-aligned cap and pedestal layer design to localize the fluidic channel within the strong field region, thereby enhancing light–matter interaction. The sensor achieved a high Q-factor of 62.59, sensitivity of 0.733 THz/RIU, and a FoM of 16.4. Despite its excellent performance compared to previous designs, the reliance on precise microfabrication for alignment may limit scalability for large-scale applications. Mahfuz *et al.* presented in paper [12], a highly simplified terahertz metamaterial absorber using L-shaped gold patches achieves 96% absorption at 1.672 THz with a Q-factor of 28.26 and demonstrates strong polarization independence. With a sensitivity of 374 GHz/RIU across the RI range of 1.304–1.342, it is highly suitable for biomedical. Study in [13] by M. S. Shoshi *et al.* introduces an enhanced SPR biosensor incorporating a hybrid BlueP/TMDCs and BaTiO_3_ multilayer structure was introduced to detect glucose concentrations in urine, achieving a high sensitivity of 435 deg/RIU, a quality factor of 86.29 RIU^−1^, and detection accuracy of 0.190 deg^−1^; unlike conventional 2D or single-layer sensors, this design improved light–matter interaction and field distribution, though its practical validation remains reliant on numerical methods alone. Another study by Jain *et al.* [14] reported, a compact hepta-band THz metamaterial absorber with T-shaped resonators achieved a peak sensitivity of 4.72 THz/RIU, while an ERT model reduced simulation time by 60% [5], though experimental validation remains limited. In biomedical applications, R. Rahad *et al.* [15] proposed a nanosensor that utilizes a metal insulator metal structure with a silver nanorod resonator, achieving ultra-high sensitivity (2963.73 nm/RIU) and high precision, enabling effective detection of glucose levels, hemoglobin concentration, early cancer diagnosis, and potential identification of biological components in brain and blood tissues. Later, in [16] M. A. Haque *et al.* introduced a plasmonic sensor achieved 2527.6 nm/RIU sensitivity and detected honey quality and lactose concentration, showing promise for biomedical sensing. In a simple and highly sensitive metasurface absorber using a bow-tie metallic patch achieves dual-band absorption at 1.93 THz and 2.7 THz, showing strong sensitivity to RI changes. It offers a peak sensitivity of 2.37 THz/RIU, Q-factor of 574.46, and FoM of 540 RIU^−1^, suitable for tuberculosis detection in blood samples, as reported in paper R. Agrahari *et al.* [17]. M. M. A. Mia *et al.* in paper [18], suggested a biomedical included model for a triple-band metamaterial absorber with concentric square copper ring resonators on an FR-4 substrate achieved near-perfect absorption of 99.99% at 0.717 and 3.535 THz, and 99.95% at 1.368 THz, optimized via a genetic algorithm for enhanced sensitivity, reaching up to 479.5 GHz/RIU. Respective design of N. Singh *et al.* offered an ultra-sensitive narrowband metamaterial biosensor, as, achieves 96.9% absorption at 2.054 THz with a sensitivity of 5002 GHz/RIU for cancer cell detection, though its performance may degrade outside the 1.0–3.0 THz range and under extreme structural deformation [19]. A dual-band THz metamaterial sensor designed L. Darsi *et al.* in [20], featuring a split ring and inverted Z-shaped unit cell, achieves unity absorption at 0.75 THz and 1.01 THz with 2.075 THz/RIU sensitivity, though its narrow RI range (1.35–1.40) limits broader applicability dependence on precise fabrication may limit practical deployment. However, its relatively narrow refractive index range and dependence on precise fabrication may limit practical deployment in more complex biological environments. Narrow absorption bandwidth enhances the quality (Q) factor and Figure of Merit (FoM) in metamaterial sensors, crucial for sensitive molecular detection. While increasing sensitivity or reducing bandwidth can improve FoM, prioritizing bandwidth reduction yields higher Q-factors and better performance. Additionally, polarization-insensitive designs ensure consistent absorption regardless of wave orientation, simplifying practical use referred in [21] introduces a multiband metamaterial biosensor operating in the 0.5–5 THz range, achieving multiple resonance bands (>8 bands) with absorption exceeding 99% and a reported high sensitivity on the order of ~10⁶ THz/RIU. Although this design shows extremely high sensitivity, it relies on a more complex multilayer configuration and extensive structural optimization. In contrast, the proposed structure achieves comparable high absorption and sensing capability using a simpler and compact geometry, improving fabrication feasibility. In lower THz regime S. M. A. Haque et al. presented a triple-band terahertz metamaterial absorber operating in the 6–8 THz range with near-unity absorption (~99.8%), a high quality factor (Q ≈ 494.3), and a sensitivity of 1.08 THz/RIU, which is more favorable for practical biosensing applications due to lower material losses and improved penetration depth in biological media [22]. Whether K. S. L. Al Badri et al. in [23], proposed multi-band metamaterial absorber exhibiting broadband absorption behavior in the microwave regime (GHz range). While this work demonstrates effective absorption (>95%) beyond the terahertz regime and is primarily focused on electromagnetic interference (EMI) and communication applications rather than biosensing. Recent studies have reported high-performance metamaterial absorbers with high Q-factor and sensitivity, particularly in the terahertz regime. While some designs operate at higher frequencies or employ complex multilayer structures, the proposed absorber achieves multi-band near-unity absorption with a compact single-layer configuration in a THz range.

Compared to microwave-based absorbers, the present design is more suitable for biosensing applications due to its operation in the terahertz domain. In this work, the novelty lies in the synergistic combination of a geometrically decoupled snowflake-ring resonator and Genetic Algorithm (GA) optimized design of triple-band terahertz (THz) metamaterial absorber, combining all relevant parameters at their optimum levels for high-resolution detection of disease-specific biomarkers. This study establishes a computational proof-of-concept framework for terahertz biosensing using a metamaterial absorber, aimed at evaluating sensing feasibility through numerical simulations and literature-based dielectric modeling, enabling systematic evaluation of sensing feasibility and performance. The proposed optimization strategy further facilitates rapid prototyping, real-time tunability, and efficient exploration of complex metamaterial architectures with significantly reduced computational cost. The proposed absorber, comprising a copper layer patterned on an FR-4 dielectric substrate, supports three distinct resonant modes at 0.925 THz, 1.71 THz, and 2.768 THz, each exhibiting near-unity absorption (99.92%, 99.97%, and 99.58%) across all bands, minimizing signal loss and improving detection accuracy. By providing three independent sensing channels, the design enables robust cross-verification of refractive index shifts, potentially improving the sensing reliability for complex biological analytes. These sharp, polarization-insensitive resonances are characterized by quality factors (Q-factors) and strong spectral selectivity achieved through systematic parametric optimization combining full-wave electromagnetic simulations. The absorber’s refractive index sensitivity, validated via rigorous numerical analysis, enables precise identification of dielectric perturbations caused by biological targets such as viral particles, cancerous cells, glucose variations, malaria parasites, and cerebral or blood components. Comparative benchmarking against existing THz sensors demonstrates a well-balanced combination of high sensitivity (10,142.86 GHz/RIU), narrow FWHM (0.078), and moderate Q-factors (~12) across all bands. Lower FWHM values indicate, as they correspond to sharper resonances and improved resolution. Emphasize the polarization insensitivity over a wide angular range (0–90°), which enhances usability in practical settings where orientation cannot always be controlled. This work advances the frontier of computational photonics by bridging data-driven optimization with metamaterial engineering. The resultant platform holds transformative potential for non-invasive, real-time diagnostics, particularly in early detection of malignancies, infectious diseases, and metabolic disorders. By enabling differentiation of healthy and pathological tissues at subwavelength scales, the sensor addresses critical unmet needs in personalized medicine and point-of-care testing.

## 2. Sensor functionality

**Fig 1:**
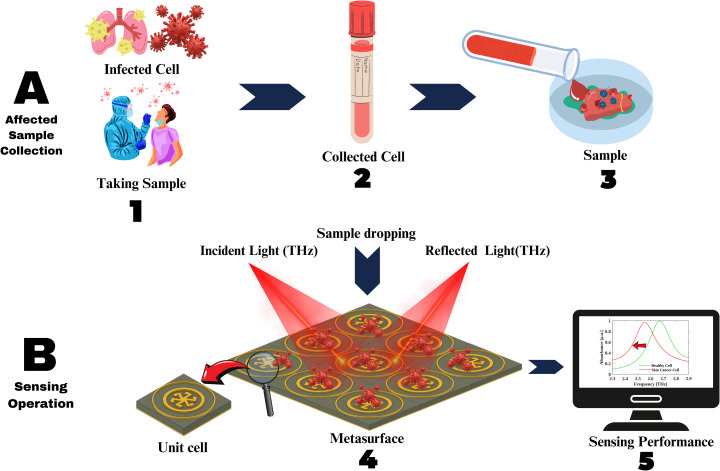
Proposed terahertz metamaterial biosensing mechanism and simulation framework.

The illustrated design methodology in Fig 1 outlines the process of terahertz (THz) metamaterial absorber for high-sensitivity biomarker detection. In the first stage (A), biological samples are collected from infected cells, processed, and prepared for analysis. In the second stage (B), a small amount of the prepared sample is deposited onto the surface of a specially engineered metasurface absorber composed of periodic unit cells, which is designed to be placed in direct contact with the sample (e.g., blood, saliva, or air). When THz radiation is directed onto the metasurface, the presence of the biomarker alters the interaction between the incident light and the metasurface, resulting in changes in the reflected THz signal. The resonance frequency (f_r_) shifts in the THz frequency due to the biomolecule’s interaction with the metasurface can be detected using THz spectrometers or THz time-domain spectroscopy (THz-TDS) systems. These changes are then analyzed and displayed as sensing performance data, allowing for the precise detection and identification of disease-specific biomarkers. This methodology utilizes the strong field confinement and resonance sensitivity of THz metamaterial absorbers to achieve rapid, label-free, and highly selective biosensing [24]. This sensing methodology illustrated in Fig 1 represents a conceptual numerical framework intended to demonstrate the operating principle of the proposed metasurface absorber. The present study does not claim experimental clinical implementation; rather, it establishes a simulation-based proof-of-concept for future terahertz biomedical sensing systems.

## 3. Design analysis

### 3.1 Design of unit cell model

The proposed structure is classified as a metamaterial absorber because its electromagnetic behavior originates from artificially engineered subwavelength resonator elements rather than intrinsic material properties. The unit-cell dimensions are substantially smaller than the operating terahertz wavelengths, allowing the periodic structure to behave as an effective electromagnetic medium. The patterned metallic resonators support localized electric and magnetic resonances that are absent in naturally occurring materials, enabling engineered control of effective permittivity and permeability. The combined excitation of electric and magnetic resonances produces impedance matching with free space while the metallic ground plane suppresses transmission, resulting in near-unity absorption. Furthermore, the observed field localization and anti-parallel surface-current distributions confirm the excitation of characteristic metamaterial resonance modes responsible for the absorber’s multiband terahertz response.

**Fig 2:**
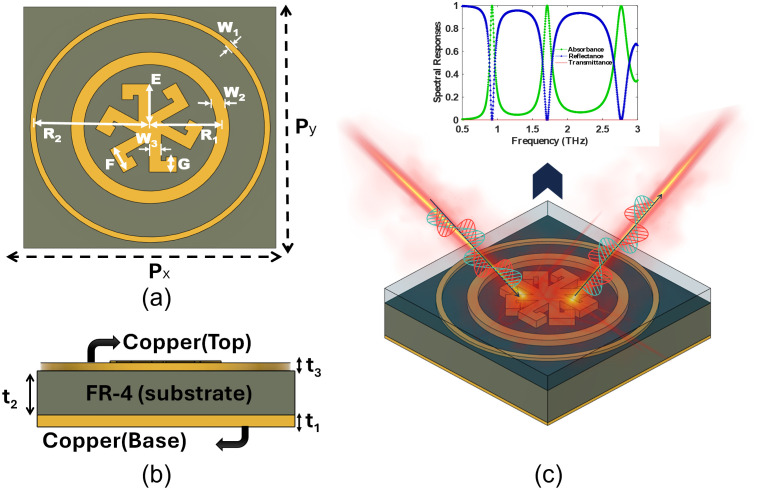
Structural overview of the proposed metamaterial unit cell. **(a)** Top view showing geometric dimensions; **(b)** cross-sectional view illustrating the copper–FR-4–copper layered structure; and **(c)** 3D schematic depicting the interaction of incident terahertz waves with the unit cell.

The design process involved iterative evaluation of multiple substrate materials, including MgF_2_, FR-4, and Fused Silica, to identify the optimal configuration. The finalized design employs an FR-4 substrate with dimension of 55 × 55 × 6.9 μm³. Both the top and bottom layers consist of copper layer thickness of t_1_ = 0.22 and t_3_ = 0.33 μm respectively illustrated in Fig 2. The top layer in Fig 2(a) comprises six central 7-shaped resonators, each separated by 60° intervals, flanked by two circular copper elements. This nested pattern defines the sensing region where analytes are introduced for performance assessment. Fig 2(a) presents the top view of a periodic square unit cell featuring a complex copper resonator pattern. This pattern includes concentric rings and symmetric arms (labeled with parameters such as R_1_, R_2_, W_1_, W_2_, W_3_, E, F, G) which are precisely engineered to support multiple resonant modes at specific THz frequencies. The geometric parameters (P_x_, P_y_) define the periodicity of the array, while the detailed features of the resonator enable strong electromagnetic coupling and field confinement, crucial for achieving high absorption at targeted frequencies. The cross-sectional view in Fig 2(b) reveals a typical sandwich structure: a patterned copper layer on top, an FR-4 dielectric substrate in the middle, and a continuous copper ground plane at the bottom. The thickness (t_1_, t_2_, t_3_) of each layer in Fig 2(b) are optimized to ensure impedance matching with free space and to suppress transmission, so that incident THz waves are either absorbed or reflected, not transmitted. 3D schematic of Fig 2(c) demonstrates how incident THz waves interact with the metasurface. The electromagnetic waves are absorbed by the resonant structure, as indicated by the strong field localization within the unit cell. The inset graph shows the absorption spectrum, highlighting multiple sharp resonant peaks corresponding to near-unity absorption at specific THz frequencies. These resonances are a direct result of engineered geometry and material composition. The optimized geometrical parameters are summarized in Table 1 and illustrated in Fig 2(a) and 2(b). Further parametric analyses and design optimizations are detailed in the following sections.

**Table 1:** Optimized design parameters of the proposed design.

Parameters	Values (µm)	Parameters	Values (µm)
Unit cell size, **P**_x_ **= P**_y_	55	Width of the 7-shaped arm, **W**_**3**_	2.5
Thickness of ground layer, **t**_1_	0.22	Center radius of first ring, **R**_**1**_	16
Thickness of dielectric layer, **t**_2_	6.9	Center radius of second ring, **R**_**2**_	25.5
Thickness of the metallic patches, **t**_3_	0.33	Seven shape, **E**	10
Width of the first ring, **W**_1_	1.05	Seven shape, **F**	6
Width of the second ring, **W**_2_	2.65	Seven shape, **G**	3.5

The electromagnetic performance of the proposed triple-band metamaterial absorber was investigated using CST Studio Suite 2022 software package through Finite-Difference Time-Domain (FDTD) method. To model a periodic array of unit cells, periodic boundary conditions were applied along the x and y axes (Floquet ports) to model the infinite metasurface array, while open boundary conditions were employed along the z-axis (the direction of wave propagation) to eliminate unwanted reflections and simulate free-space propagation. Copper element was modeled using the Drude model with plasma frequency ω_p_ = 13.4 × 10¹⁵ s^−1^ and collision frequency ν_c_ = 0.14 × 10¹⁵ s^−1^. The FR-4 substrate was modeled with a relative permittivity of ε_r_ = 4.3 and loss tangent tan δ = 0.025. Mesh refinement was applied at the metallic interfaces and gap regions to ensure convergence, with a maximum mesh cell size of λ/20 at the highest frequency of interest.

### 3.2 Performance parameter tuning and analysis

**Fig 3:**
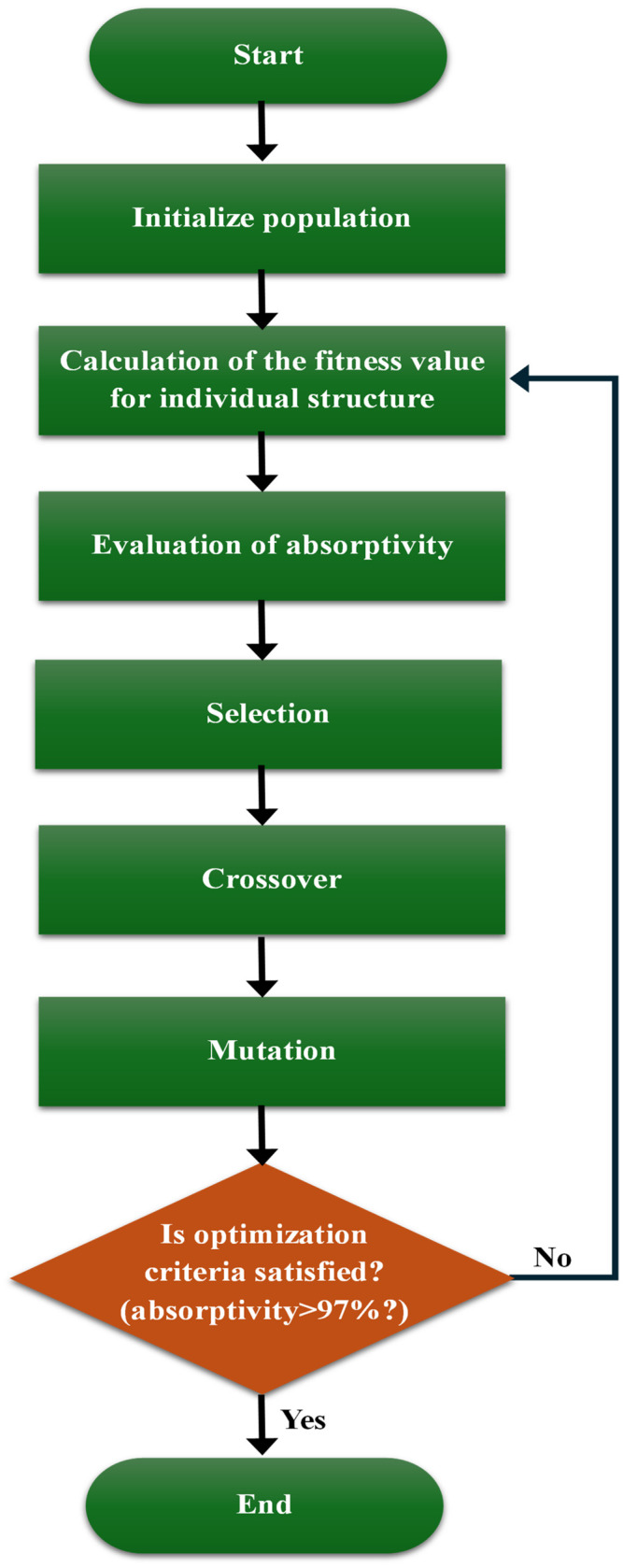
Flowchart of the genetic algorithm based on proposed model.


The sensing performance metrics, including absorbance, sensitivity, and Figure of Merit (FoM), are intrinsically linked to the resonator’s geometric configuration and performance. Traditional parameters sweep methods often fail to identify global optima due to the complex electromagnetic response of metamaterial absorbers. To overcome this challenge, genetic algorithms (GA) offer an efficient approach for optimizing the geometric parameters of terahertz metamaterial absorbers, addressing the nonlinear and multimodal nature of their design space. Implementation of GA in Fig 3 initializes a population of 100 candidate designs, each encoded with key geometric variables. Full-wave electromagnetic simulations evaluate fitness based on absorptivity and bandwidth. Through iterative processes involving selection, crossover, and mutation over approximately 150 generations, the GA converges toward optimal configurations. The fitness function used in our GA is based on the average absorptivity of the metamaterial absorber (MA) across a specified frequency range. Specifically, it calculates the average absorptivity at frequencies where the absorptivity exceeds 96%, thereby favoring solutions with broad high-absorption bandwidths. Fitness function *F* is given by:

$$\begin{array}{c} F=\frac{\text{1}}{N}\sum_{i=\text{1}}^{N}A\left(f_{i}\right)\text{ where }A\left(f_{i}\right)\geq \text{0}.\text{9}\text{7} \end{array}$$

Here, $A\left(f_{i}\right)$ represents the absorptivity at frequency $f_{i}$, and *N* is the number of frequency points where $A\left(f_{i}\right)\geq \text{0}.\text{9}\text{7}$. This approach ensures that GA favors solutions with both high absorptivity and wideband performance. Encoding geometric parameters as genes each candidate solution in the GA represents a vector (or “chromosome”). The fitness function is formulated to maximize absorptivity while satisfying design constraints such as operational bandwidth, angular stability, and fabrication feasibility. For terahertz absorber applications, the target absorptivity threshold is typically set above 99% to ensure near-perfect absorption characteristics. This method effectively balances exploration and exploitation, enabling discovery of high-performance absorber geometries that surpass traditional design approaches, while ensuring computational efficiency and robustness against local optima. This integrated approach enables more comprehensive design exploration, leading to enhanced absorber performance.

The electromagnetic absorption behavior of the proposed metamaterial absorber can be quantitatively described using scattering parameters (S-parameters). The absorptance A(ω) is expressed as:


$$\begin{array}{c} A(\omega )=\text{1}-R(\omega )-T(\omega ) \end{array}$$

where the reflectance and transmittance are respectively defined as:


$$\begin{array}{c} R(\omega )=|S_{\text{1}\text{1}}{|}^{\text{2}} \end{array}$$

and


$$\begin{array}{c} T(\omega )=|S_{\text{2}\text{1}}{|}^{\text{2}} \end{array}$$

Since the proposed structure incorporates a continuous metallic ground plane, the transmission through the structure is effectively suppressed (T(ω)≈0). Therefore, the absorption can be simplified as:


$$\begin{array}{c} A(\omega )=\text{1}-|S_{\text{11}}{|}^{\text{2}} \end{array}$$

The absorption mechanism can further be interpreted using an equivalent LC resonance model. Each metallic ring resonator behaves as an effective inductance-capacitance (LC) circuit, where the metallic loop contributes inductance L, while the capacitive gaps between adjacent metallic regions generate capacitance C. The resonance frequency of the absorber follows:


$$\begin{array}{c} f_{res}=\frac{\text{1}}{\text{2}\pi \sqrt{LC}} \end{array}$$

Variations in the dielectric properties of surrounding analytes alter the effective capacitance of the resonator system, resulting in measurable resonance-frequency shifts. This behavior forms the basis of refractive-index-based biosensing.

To evaluate sensing performance quantitatively, the sensitivity S, quality factor Q, and Figure of Merit (FoM) are calculated using:


$$\begin{array}{c} S=\frac{\Delta f}{\Delta n} \end{array}$$

$$\begin{array}{c} Q=\frac{f_{res}}{FWHM} \end{array}$$

$$\begin{array}{c} FoM=\frac{S}{FWHM} \end{array}$$

where $f_{res}$ and Δf denotes the resonance-frequency and shift of resonance frequency caused by refractive-index variation Δn respectively, and FWHM represents the full width at half maximum of the resonance peak. Higher values of sensitivity (S), Q-factor (Q), and FoM indicate enhanced sensing resolution and spectral selectivity.

**Fig 4:**
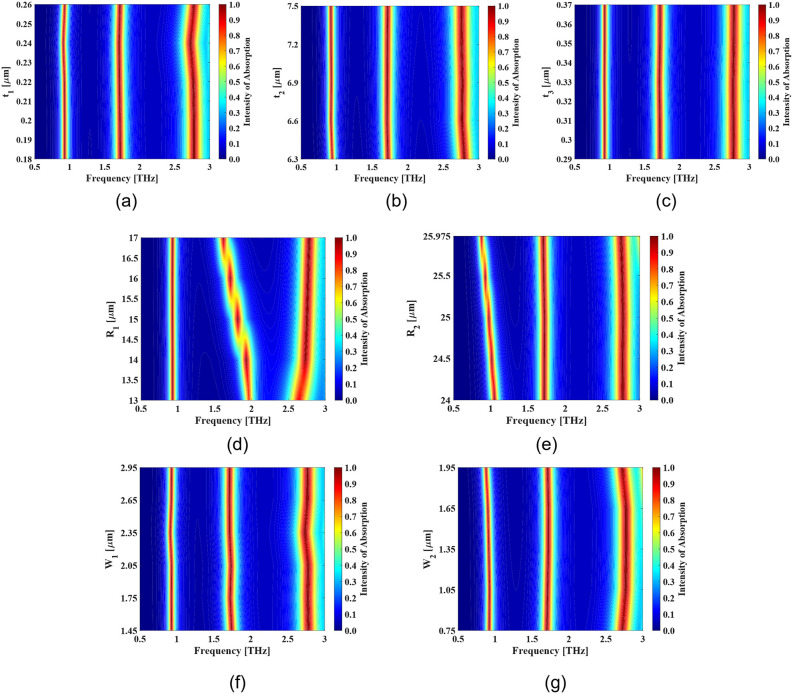
Parametric optimization of the proposed metamaterial absorber. **(a–c)** Effect of varying layer thickness (t_1_, t_2_, t_3_) on resonance frequency shifts; **(d–e)** influence of ring radius (R_1_, R_2_) variations; **(f–g)** impact of ring width (W_1_, W_2_) changes.


All optimizations were performed under a fixed refractive index (n = 1) for the sensing medium to isolate geometric effects. Each parameter was varied individually while the others were held constant to isolate their specific effects on the absorption characteristics. After applying the genetic algorithm, a systematic parametric sweep was conducted on the unit cell by varying critical dimensions, including absorber thickness (t_1_, t_2_, and t_3_), radius (R_1_ and R_2_), and width (W_1_ and W_2_), while iteratively evaluating their impact on the electromagnetic response. Fig 4 presents a detailed parametric study of the proposed terahertz metamaterial absorber, illustrating how variations in these key geometric parameters influence their absorption and reflection characteristics across the terahertz frequency range. Fig 4(a–c) presents color plots illustrating the effects of varying structural parameters on the spectral responses of the metamaterial absorber, with each plot focusing on distinct parameters; notably, thickness optimization is critical for achieving optimal impedance matching, directly influencing absorbance characteristics and overall absorption efficiency. The impact of varying the thicknesses of different layers, denoted as t_1_ (0.18–0.26 μm), t_2_ (6.3–7.5 μm) and t_3_ (0.29–0.37 μm) on the absorbance response. Changes in dielectric layer thickness primarily affect the impedance matching condition between the absorber and free space. The periodicity was fixed during single-parameter sweeps to avoid coupling effects. Through this iterative tuning process, the final design achieved near-unity absorption (99.92%, 99.97%, and 99.58%) at targeted terahertz resonance frequencies (0.925, 1.71, and 2.768 THz) with enhanced performance and confirming the effectiveness of the parametric optimization approach for tailoring the metamaterial absorber’s performance when t_1_ = 0.22 μm, t_2_ = 6.9 μm and t_3_ = 0.33 μm. Thinner layers tend to broaden the resonance and slightly reduce the depth of the S_11_ minima, while thicker layers sharpen the resonance and deepen the S_11_ dip, signifying improved absorption efficiency. The results underscore the importance of precise thickness control for optimizing both bandwidth and peak absorption. The electromagnetic penetration depth inside the metallic resonator can be estimated using the skin depth equation:

$$\begin{array}{c} \delta =\sqrt{\frac{\text{2}}{\omega \mu \sigma }} \end{array}$$

where δ is the skin depth, ω is the angular frequency, μ is the magnetic permeability, and σ is the electrical conductivity of copper. At terahertz frequencies, the calculated skin depth is significantly smaller than the thickness of the deposited copper layers, ensuring negligible electromagnetic transmission through the metal and contributing to near-unity absorption performance.

The effects of varying the radius R_1_ and R_2_ of concentric circular resonator elements were investigated in Fig 4(d) and 4(e). An increase in radius R_1_ induces a red-shift in the second resonance band, attributed to enhanced capacitive effects and modifications in current distribution within the resonator. Conversely, increasing radius R_2_ results in a red-shift of the first resonance band. Peak absorbance values of 99.94%, 99.78%, and 99.45% were observed at R_1_ = 16 μm, while values of 99.99%, 99.84%, and 99.15% were recorded at R_2_ = 25.5 μm, respectively. These results indicate that the absorber’s performance remains robust against moderate variations in geometrical parameters, which is advantageous for fabrication tolerance. Absorbance spectra corresponding to varying resonator widths W_1_ and W_2_ are presented in Fig 4(f) and 4(g). An increase in W_1_ induces shifting of the resonance peak, whereas an increase in W_2_ causes a red-shift in the corresponding resonance band. Notably, the absorption magnitude remains high (>99.9%) across all variations, with optimal performance observed at W_1_ = 2.65 μm and W_2_ = 1.05 μm, indicating strong coupling between the structure and incident terahertz waves. This spectral tunability is essential for optimizing the absorber for targeted applications such as selective sensing and filtering.

**Fig 5:**
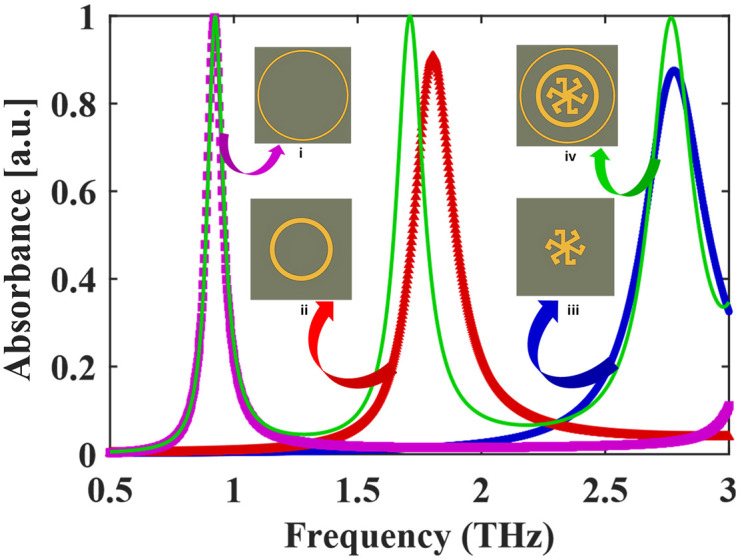
Absorption spectra for four internal resonator configurations demonstrating performance robustness.

The absorbance spectrum of the optimized structure, shown in Fig 5, exhibits three distinct spectral responses (dips) corresponding to structure (i-iv). It was observed that the absorbance of these resonance peaks remained above 87% across variations in individual structure, demonstrating robustness in absorption intensity, i.e., when only the outer ring is present, the resonant frequency is higher compared to configurations including the middle and center elements. However, the resonance frequencies are sensitive to changes in the geometric dimensions; specifically shape of circular width and radius caused a number of absorption peaks. Structure iv demonstrates three distinct absorption peaks in Fig 5 at 0.925 THz, 1.71 THz, and 2.768 THz, with peak absorptances of 99.92%, 99.97%, and 99.58%, respectively. This multi-band response contrasts with the singular resonant band observed in other structures (i-iii), highlighting its superior potential for multifrequency sensing applications.

**Fig 6:**
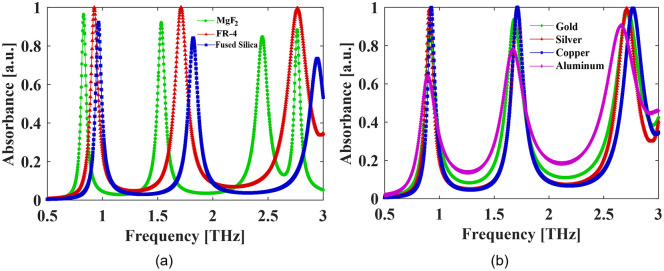
Comparison with (a) substrate and (b) radiating materials on absorption characteristics.

Fig 6(a) illustrates three common substrates magnesium fluoride (MgF_2_), FR-4, and Fused Silica to evaluate the effect of dielectric variation on absorption performance. The meticulous optimization of substrate materials is paramount in engineering terahertz (THz) metamaterial absorbers with superior performance. As demonstrated in Fig 6(a), the absorber’s spectral response exhibits profound sensitivity to the dielectric properties of the substrate. Utilizing magnesium fluoride (MgF_2_) as the substrate material yields exceptionally sharp and intense absorption resonances at elevated frequencies, signifying optimal electromagnetic confinement and minimal dielectric losses.

In contrast, the employment of FR-4 introduces a deliberate blue-shift in resonance accompanied by broadened absorption bandwidths, a direct consequence of its inherently higher permittivity and loss tangent. Fused silica substrates, while offering structural stability, manifest comparatively attenuated and broadened resonance peaks, reflecting suboptimal energy confinement and diminished absorptive efficiency. These findings unequivocally underscore the indispensable role of substrate dielectric optimization in precisely tuning resonance frequencies and maximizing absorption efficacy, thereby enabling bespoke absorber designs tailored for targeted THz applications. Table 2 presents a comparative performance analysis of different substrate materials, highlighting their impact on the no. of bands, absorption efficiency, and resonance of the metamaterial absorber. To address the inherent losses of FR-4, the absorber structure was carefully engineered through choice of geometry and field localization techniques. Specifically, field confinement and enhancement were achieved by optimizing the resonant structures, which are designed to minimize the influence of the lossy substrate on key performance metrics such as absorption, reflection, and transmission. These strategies collectively mitigate the adverse effects of dielectric loss and enable the absorber to maintain near-perfect absorption at the target frequencies, as demonstrated by the simulation results. It is equally critical is the strategic selection and optimization of the metallic resonator constituent, as depicted in Fig 6(b) and properties in Table 3 [25] that shows variations in material properties significantly affect the resonant behavior of the proposed structure. Metals such as silver and copper, distinguished by their superior electrical conductivity and minimal intrinsic losses at THz frequencies, facilitate the realization of absorption peaks with unparalleled intensity and spectral sharpness. Gold, while exhibiting commendable plasmonic properties, yields broader resonance features due to comparatively elevated ohmic losses, whereas aluminum’s lower conductivity culminates in significantly compromised absorption magnitude and resonance quality. Copper, due to its high electrical conductivity, exhibited the best performance with a peak absorbance of 99.97%.

**Table 2:** Performance comparison of various substrates.

*Metals*	*Number of bands*	*Resonance* *Frequency (THz)*	*Absorption* *(Avg %)*
MgF_2_	3	0.827, 1.532, 2.447	90.41
FR-4	3	0.925,1.71, 2.768	99.97
Fused Silica	3	0.962, 1.823, 2.947	80.26

**Table 3:** Material properties [25].

Metals	Plasma frequency (*ω*_*p*_ *× 10*^*15*^ *S*^*-1*^)	Collision frequency (*v*_*c*_ *× 10*^*15*^ *S*^*-1*^)
Gold	13.8	0.11
Silver	14.0	0.032
Copper	13.4	0.14
Aluminium	22.9	0.92

This significant difference highlights the critical importance of selecting the appropriate metal to maximize quality factors and absorption efficiency, ultimately enhancing the performance of THz metamaterial absorbers. Impact of conductive materials are summarized in Table 4. The rigorous optimization of both substrate and metallic components emerges as a decisive factor in dictating the absorber’s resonance precision, absorption magnitude, and bandwidth control. This dual-material optimization framework is foundational for advancing the design of THz metamaterial absorbers, empowering the development of highly efficient, tunable, and application-specific devices for next-generation sensing, imaging, and communication technologies. For bio-THz sensors, one often requires microfluidics or sample handling on the metamaterial. Substrates like fused silica can be integrated with microfluidic channels (etched glass) without interfering with THz performance, whereas FR-4 is hydrophilic and may swell. Metallic choices also affect integration: Au surfaces allow straightforward thiol-linker chemistry to bind antibodies or analytes, whereas non-gold metals require different surface treatments.

**Table 4:** Performance evaluation of different conductive materials.

*Metals*	*Number of bands*	*Resonance Frequency (THz)*	*Absorption* *(Avg%)*
Glod	3	0.912, 1.683, 2.72	93.61
Silver	3	0.907, 1.71, 2.71	99.31
Copper	3	0.925, 1.71, 2.768	99.97
Aluminum	3	0.892, 1.67, 2.658	77.57

When combined with an FR-4 substrate, the copper-based design achieved three distinct absorption peaks at 0.925, 1.71, and 2.768 THz with a resonator thickness of t_3_ = 0.33 μm, underscoring the critical role of material selection in optimizing terahertz absorber performance. This FR-4, Cu-based designs offer simplicity and cost-effectiveness; however, the study emphasizes how variations in substrate dielectric properties influence resonance behavior and overall absorption efficiency, thereby guiding optimal substrate selection for enhanced device performance in terahertz sensing applications.

### 3.3 Cell bending properties and polarization

**Fig 7:**
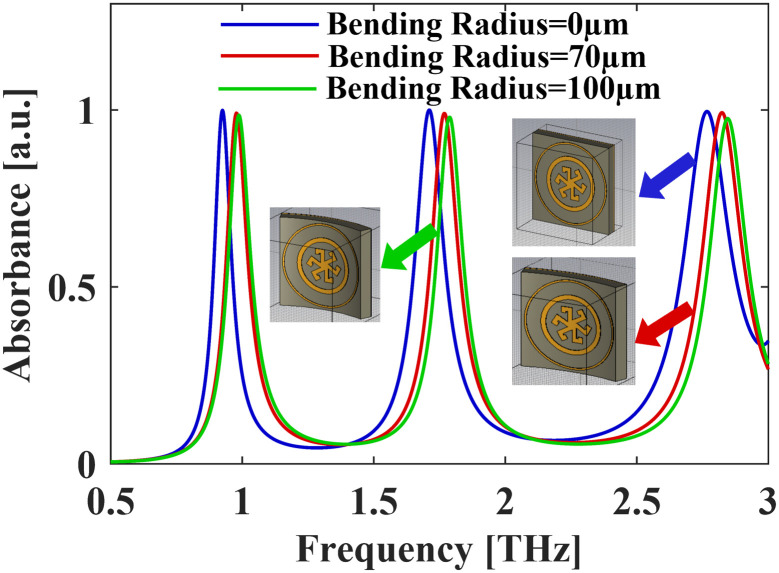
Effect of Resonance stability under varying structural bending conditions.

Fig 7 illustrates the absorbance spectra of the terahertz metamaterial absorber under different bending radii: 0 μm (flat), 70 μm (moderate bending), and 100 μm (high bending). The results clearly show that the absorber maintains high absorbance at all three resonance peaks across the frequency range of 0.5–3 THz, even under significant mechanical deformation. However, subtle yet important shifts in both resonance frequency and peak intensity are observed as the bending radius increases. Specifically, as the bending radius increases, the resonance peaks exhibit a slight red-shift (shift to lower frequencies) and a minor reduction in peak absorbance. This effect is most pronounced at the higher-frequency modes, as indicated by the colored arrows in the Fig 7. The primary cause of these spectral changes is the mechanical strain induced by bending, which alters the effective geometry and spacing of the metamaterial’s resonator elements. Bending can increase the physical distance between unit cells on the outer surface while compressing those on the inner surface, thereby modifying the local electromagnetic coupling and effective path lengths. This geometric distortion leads to a decrease in the resonance frequency (blue-shift) and can introduce additional scattering or loss, slightly reducing the maximum absorbance. Despite these changes, the absorber demonstrates robust performance and retains near-unity absorbance at all resonance modes, underscoring its mechanical flexibility and suitability for applications in flexible or conformal terahertz devices. The minimal impact of bending on the absorber’s spectral response highlights the effectiveness of the design in maintaining functional integrity under mechanical deformation, which is essential for practical deployment in wearable sensors, flexible imaging arrays, and other advanced THz technologies.

**Fig 8:**
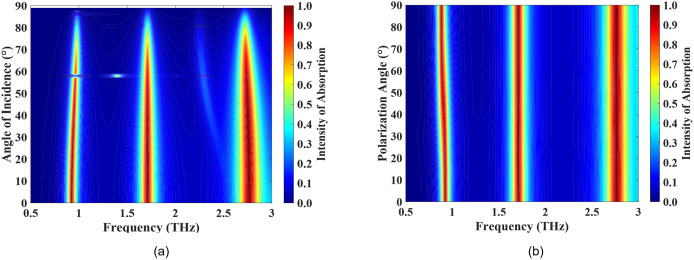
Absorption performance of the proposed terahertz metamaterial absorber. **(a)** Incident and **(b)** polarization angles across 0.5–3 THz, showing strong resonance peaks with absorption >0.9 up to ~60° incidence, maintaining effective response across a wide angular range.

Fig 8 presents a comprehensive analysis of the absorption characteristics of the proposed terahertz metamaterial absorber as a function of both incident angle (Fig 8a) and polarization angle (Fig 8b) across the frequency range of 0.5–3 THz. The color plot in Fig 8(a) illustrates the variation in absorption intensity with respect to different angles of incidence, ranging from 0° to 90°. The results indicate that the primary absorption peaks, especially those near 0.925 THz, 1.71 THz, and 2.768 THz, remain highly robust for incident angles up to approximately 60°. The intensity of absorption at these resonant frequencies consistently exceeds 0.9, as shown by the persistent red bands. However, as the angle of incidence approaches 70° and beyond, a gradual decrease in absorption intensity is observed, particularly for the lower-frequency modes. This behavior can be attributed to the increased reflection and reduced coupling efficiency at high incident angles, which is a common phenomenon in planar metamaterial absorbers. The observed angular stability up to large incident angles underscores the practical applicability of the absorber in scenarios where the direction of incoming terahertz waves may vary, such as in imaging or sensing applications requiring wide angular coverage. Fig 8(b) depicts the absorption intensity as a function of polarization angle, from 0° to 90°. Remarkably, the absorption profile exhibits minimal variation across the entire polarization range for all major resonance frequencies. The vertical red bands, which indicate high absorption, remain continuous and uniform regardless of the polarization state. The maximum variation in peak absorption across polarization angles 0°–90° is less than 0.5% at all three resonance frequencies, confirming complete polarization insensitivity. This polarization insensitivity is a direct consequence of the absorber’s symmetric unit cell design, which ensures that the resonant modes are equally excited by both Transverse Electric (TE) and Transverse Magnetic (TM) polarized waves. Such polarization-independent behavior is highly desirable for practical terahertz devices, as it guarantees consistent performance regardless of the polarization orientation of the incident radiation. For oblique incidence, the plane of incidence is defined by the incident wave vector and the surface normal of the metasurface. In TE polarization, the electric field is perpendicular to the plane of incidence, whereas in TM polarization, the magnetic field is perpendicular to the plane of incidence. Under normal incidence, both polarization states exhibit equivalent electromagnetic behavior due to the symmetry of the structure. The combined results from both panels demonstrate that the proposed metamaterial absorber exhibits excellent angular and polarization stability. The high absorption efficiency maintained over a broad range of incidents and polarization angles highlights the robustness and versatility of the design. This makes the absorber particularly suitable for real-world terahertz applications, including broadband sensing, imaging, and stealth technologies, where environmental and operational conditions may not be precisely controlled. Optimal angular configurations enhance signal fidelity by maximizing absorption efficiency and minimizing noise. The observed angular robustness, along with stable SNR characteristics, underscores the biosensor’s potential for reliable terahertz imaging, offering promising applicability in the early detection of cancer and other biomedical diagnostics.

### 3.4 EM field and surface current distribution

**Fig 9:**
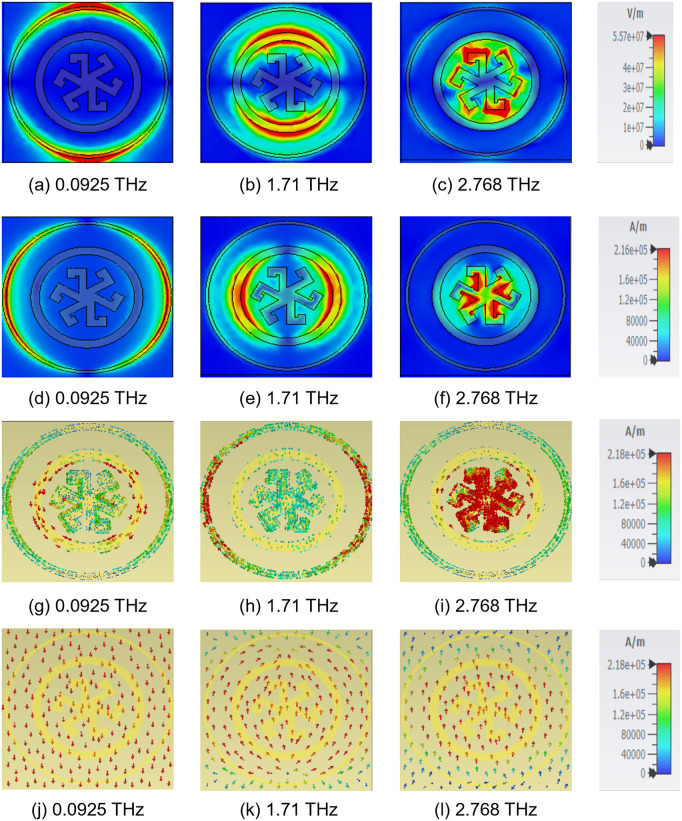
Electric field, magnetic field, and surface current distributions at resonance frequencies. **(a–c)** Electric field (E-field) distributions, **(d–f)** magnetic field (H-field) distributions, and **(g-l)** surface current distributions of top and bottom layer of the proposed unit cell at its resonance frequencies, highlighting the electromagnetic behavior and current flow patterns responsible for the metamaterial’s absorption performance.

To investigate the absorption behavior of the proposed terahertz metamaterial absorber, detailed analyses of the electric (E-field) and magnetic (H-field) field distributions were performed at the three principal resonance frequencies of 0.925 THz, 1.71 THz, and 2.768 THz, as shown in Fig 9(a–c) and Fig 9(d–f). The E-field profiles demonstrate a progressive confinement of the maximum electric field intensity (2.52 × 10^7^ V/m), shifting vertically from the outer boundaries first peak toward the central circular region with increasing frequency to third peak. Correspondingly, the H-field distributions reveal enhanced magnetic field localization near the edges of the resonator structure, indicative of strong electromagnetic confinement horizontally and resonance enhancement. This pronounced localization arises primarily from the geometric asymmetry and subwavelength scale of the resonator elements, which facilitate the excitation of localized surface plasmon resonances and induce strong capacitive coupling across the gap regions. Such coupling leads to intense near-field enhancement and spatial confinement of electromagnetic energy (max. 133573 A/m) within the unit cell. The inward shift of field confinement across increasing resonance frequencies is a result of the resonant modes being supported by progressively smaller structural features of the metamaterial. At lower frequencies, the longer wavelengths interact more effectively with the larger outer boundary structures, leading to strong field localization in those regions. As the resonance frequency increases, the effective wavelength becomes shorter, enabling the excitation of higher-order modes that couple more efficiently with smaller features, such as the intermediate and innermost square boundaries. This spatial progression of field localization supports the multiband absorption behavior of the metamaterial by enabling each structural scale within the resonator to support a distinct resonance mode. The outer, intermediate, and inner geometrical elements act as independent resonators tuned to different frequencies, contributing to multiple absorption peaks. The distinct field localization at each frequency ensures minimal modal overlap and strong resonance at separate spectral bands, which is a key characteristic of efficient multiband absorbers. The sensing mechanism is governed by strong evanescent field confinement at the resonator surface. These fields extend into the analyte layer with subwavelength penetration depth, enabling high sensitivity to local dielectric perturbations. The resulting change in effective capacitance leads to measurable resonance frequency shifts.

The surface current distributions (top and bottom layer) at the same resonance frequencies, depicted in Fig 9(g–i) and Fig 9(j–l) respectively, further corroborate this behavior. On the top metallic layer Fig 9(g–i), the maximum current density is concentrated at the 7-shaped flanked central resonator. The peak current density reaches values as high as 217769 A/m for third resonance (2.74 THz). When an analyte covers the sensor surface, changes in local permittivity alter the effective capacitance, leading to measurable shifts in the resonance frequency. Additionally, dielectric losses introduced by the sample attenuate both the electromagnetic field intensity and surface current density, modulating the absorption characteristics. The absorption mechanism of the proposed metamaterial absorber is governed by coupled electric and magnetic resonances arising from the geometrically engineered resonator structure. The concentric metallic elements and distributed 7-shaped resonators support multiple localized electromagnetic eigenmodes with distinct current pathways and field confinement regions. At resonance, strong capacitive coupling occurs across the narrow metallic gaps, while circulating surface currents generate effective inductive behavior, forming localized LC resonances. This behavior is consistent with the equivalent LC resonance model discussed in Section 3.2. In Fig 9(j–l) at the first and third resonance frequencies, the current vectors on the bottom metallic layer exhibit anti-parallel vertical alignment, indicating the presence of parallel currents within the metamaterial unit cell. In contrast, the second resonance peak is characterized by a turbulent circular current flow localized at the center of the structure. The resonance frequencies are therefore determined by the equivalent inductance and capacitance of the structure. Simultaneously, anti-parallel current distributions between the top resonator and bottom metallic ground plane produce magnetic dipole resonances that contribute to impedance matching with free space. Under this condition, reflection is significantly suppressed while transmission is eliminated by the metallic ground layer, resulting in near-unity absorption. The electric-field and magnetic-field distributions shown in Fig 9 confirm strong electromagnetic localization within the resonator gaps and central regions, indicating enhanced light–matter interaction. When biological analytes are introduced onto the sensing surface, variations in the surrounding dielectric environment perturb the localized electromagnetic fields and modify the equivalent capacitance, thereby producing measurable resonance-frequency shifts that enable refractive-index-based biosensing.

### 3.5 Fabrication process

**Fig 10:**
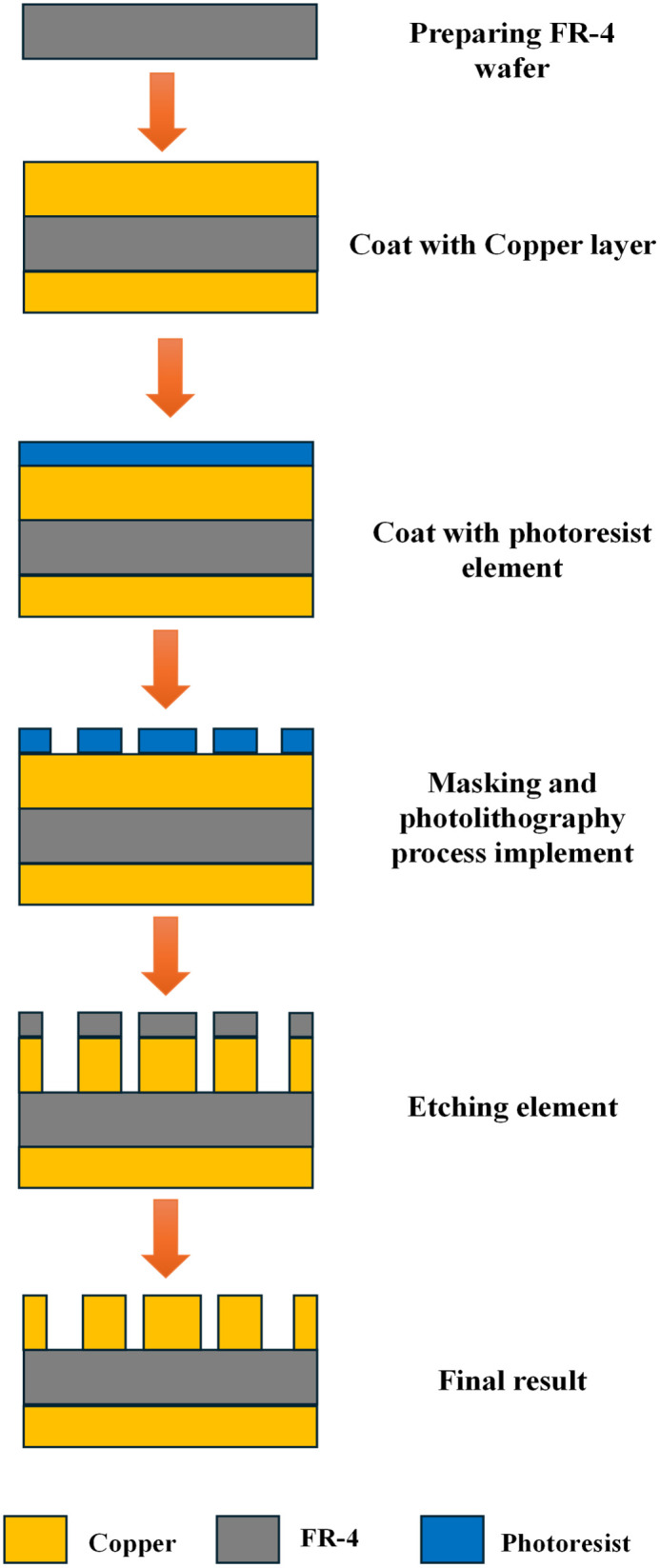
Fabrication and construction process of a plasmonic terahertz metamaterial absorber.

A FR-4 substrate of defined thickness will be selected for the fabrication of the proposed metamaterial structure. Prior to lithographic processing, the substrate will be thoroughly cleaned and dried to ensure optimal surface conditions. A thick film of copper with lateral dimensions will be applied by placing it onto the FR-4 substrate pre-treated with ethanol. Gentle pressure will be applied using a cotton swab to remove trapped air and promote adhesion by facilitating ethanol evaporation, ensuring intimate contact between the FR-4 and copper layers. Subsequently, a positive liquid photoresist will be spin-coated onto the top surface and soft-baked according to manufacturer specifications. Photolithography using a 7-shaped flanked patterned mask will be employed to define the metamaterial geometry on the photoresist. Following pattern transfer, a thin copper layer is deposited and excess material is removed via lift-off or wet chemical etching to realize the final resonator structure. The complete fabrication procedure of the proposed metamaterial absorber is illustrated in Fig 10. The residual photoresist will then be removed with acetone, leaving the patterned copper on the FR-4 substrate. The FR-4 substrate will subsequently be separated from the copper layer, and a 6.9 µm thick copper film will be deposited onto the FR-4 structure via vacuum evaporation to form a fully metallic metamaterial absorber unit cell. Finally, a periodic array of m × n unit cells will be fabricated over a micro-scale area, where m and n are positive integers. While lithography is expected to be effective for this micro or nanoscale fabrication, challenges such as high cost, process complexity, and resolution limitations will need to be addressed during development.

It should be noted that the dimensions presented in this work correspond to a single metamaterial unit cell rather than the complete sensing device. In practical implementations, the proposed structure would be fabricated as a large-area periodic metasurface array composed of thousands of repeated unit cells (m × n unit cells) over a millimeter or centimeter-scale substrate. The fabricated metasurface can be experimentally characterized using conventional terahertz time-domain spectroscopy (THz-TDS) systems or continuous-wave THz sources, where a collimated terahertz beam illuminates the entire metasurface simultaneously [26]. For biosensing applications, biological analytes are not deposited onto an individual micrometer-scale resonator; instead, the sample is introduced uniformly across the metasurface using droplet deposition, or thin-film coating techniques. The sensing mechanism relies on resonance perturbation caused by changes in the effective dielectric environment surrounding the resonators, enabling practical label-free detection of biological samples under realistic operating conditions.

## 4. Results investigation

### 4.1 Performance of unit cell

**Fig 11:**
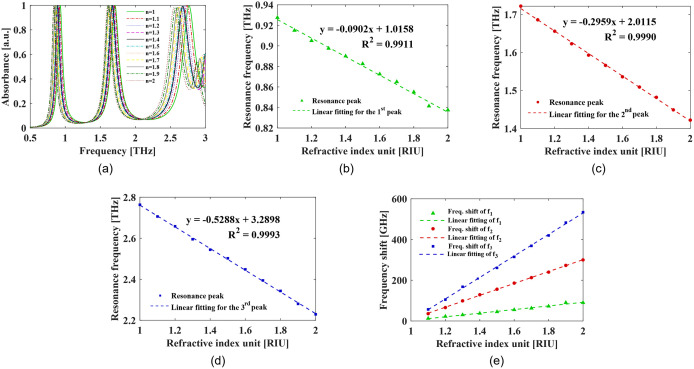
Performance of refractive-index sensing, sensitivity analysis, and resonance frequency shifts. **(a)** Absorption spectra at varying refractive indices, **(b–d)** Linear fitting and sensitivity evaluation of resonance frequency shifts for three distinct peaks, and **(e)** Frequency shifts plotted against refractive index, demonstrating strong linear correlation, confirming the absorber’s reliable sensing performance.

The three resonance frequencies generated by the proposed metamaterial structure provide important advantages for multi-band terahertz biosensing applications. The resonance frequencies at 0.925 THz, 1.71 THz, and 2.768 THz originate from distinct electromagnetic eigenmodes supported by the optimized metamaterial geometry rather than being preassigned to specific biomedical targets. Each resonance mode exhibits unique electromagnetic field localization and coupling characteristics, enabling different levels of interaction with surrounding dielectric media. Since biological analytes exhibit frequency-dependent dielectric dispersion in the terahertz region, the presence of multiple resonant modes improves spectral selectivity, sensing robustness, and discrimination capability for biomolecules with closely related refractive indices. In particular, the higher-frequency resonance demonstrates stronger field confinement and enhanced refractive index sensitivity, making it highly promising for detecting subtle dielectric perturbations associated with cancer cells, viral particles, glucose concentration changes, and intracellular biological components. This investigation establishes a framework for assessing metamaterial-based sensors through systematic analysis of resonance dynamics under control refractive index (n) perturbations. By probing the interplay between structural parameters and optical response, the study quantifies performance metrics for a triple-band absorber optimized for biomolecule analyte detection. Subsequent spectral characterization under n = 1.0–2.0 variations (Δn = 0.1) demonstrated three distinct, red-shifted resonance frequencies with amplitude stability at lower bands and gradual degradation above 2 THz in Fig 11(a). This fine-tuning step enables detection of subtle changes relevant to biomolecule sensing. Results show a red shift in all three resonance frequencies with an increasing refractive index, while absorption amplitudes remain stable, except for a gradual decrease at higher frequencies. Linear regression of frequency shifts (Δf) against n yielded sensitivities (S = Δf/Δn) of 90 GHz/RIU, 296 GHz/RIU, and 529 GHz/RIU for the respective resonance modes, supported by near-unity R² values (0.9911–0.9993) confirming strong correlation in Fig 11(b–d). Spectral resolution was further quantified via Full Width at Half Maximum (FWHM) measurements, with values of 0.078, 0.135 and 0.222 corresponding to Q-factors of 11.86–12.67 and Figure of Merit ($FoM=\frac{S}{FWHM}$) ranging from 1.154 to 2.383. Notably, the third resonance exhibited superior sensitivity (529 GHz/RIU) despite moderate change of FoM, suggesting a trade-off between detection and resolution. The corresponding Q-factor and Figure of Merit (FoM) were evaluated using the relations introduced in Section 3.2. The multi-band response enables selective sensing at different frequency ranges. Higher sensitivity modes (f_3_) provide better detection limits for small refractive index changes. Fig 11(e) shows frequency shift versus refractive index for three resonance modes (f_1_, f_2_, f_3_), highlighting key biosensing sensitivities. Each resonance exhibits a linear relationship between refractive index changes and frequency shifts, as indicated by the dashed fitting lines. The linearity confirms the absorber’s predictable response to dielectric environment changes.

Comparative analysis against existing designs confirms enhanced multi-band responsiveness, attributed to optimized metamaterial geometry that sustains >99.5% absorption while minimizing cross-mode interference. These attributes underscore the structure’s potential for integration into miniaturized sensing systems requiring simultaneous multi-analyte discrimination. A detailed summary of the performance metrics is provided in Table 5. The achievement stems from the sensor sensitivity of 529 GHz/RIU at 2.768 THz, which enhances its capacity to resolve minute dielectric perturbations caused by trace analytes. This performance aligns with advancements in THz metamaterials achieving 2.537 THz/RIU sensitivity in refractive index sensing [27].

**Table 5:** Performance summary of proposed absorber.

*Peak*	*Resonant frequency (THz)*	*Sensitivity* (GHz/RIU)	*FWHM*	*Q-Factor*	*FoM*	*Absorbance* *(%)*
1^st^ peak	0.925	90	0.078	11.8589	1.154	99.92
2^nd^ peak	1.71	296	0.135	12.6666	2.193	99.97
3^rd^ peak	2.768	529	0.222	12.4662	2.383	99.58

### 4.2 Response characterization

In this work, biomedical analytes including cancer cells, viruses, glucose solutions, blood components, intracellular materials, and biological tissues were modeled using an effective dielectric-layer approximation based on experimentally reported refractive index values available in the literature. During full-wave electromagnetic simulations, a thin analyte layer was introduced above the metasurface sensing region, and its refractive index was varied according to the corresponding biological sample. he sensing mechanism relies on resonance perturbation caused by changes in the effective dielectric environment surrounding the resonator structure. The characterization of the proposed design demonstrates enhanced sensitivity (S) and high specificity in detecting biomolecules, including those with similar refractive indices (RI), by integrating multiple complementary mechanisms. Key factors contributing to this performance include resonance frequency shifts (∆f), which respond sensitively to changes in the local environment; molecular structural differences that affect interaction with the sensing surface; variations in dielectric permittivity (ε) among biomolecules, which modulate the device’s electrical response; and the presence of multiple absorption peaks that provide distinct spectral signatures for different analytes. Additionally, the design’s high spectral resolution and sensitivity enable precise discrimination between biomolecules even when their refractive indices overlap. Collectively, these mechanisms allow the system to effectively differentiate and identify biomolecules with subtle differences, surpassing limitations inherent in refractive index-based sensing alone. This multifactorial approach aligns with recent findings that highlight the importance of dielectric constant variations and device architecture in optimizing biosensor sensitivity and selectivity [28,24].

The proposed sensor operates on bulk refractive index modulation and therefore lacks intrinsic chemical selectivity. Analytes with identical or closely matched refractive indices will produce similar resonance shifts, making them indistinguishable in the absence of selective binding mechanisms. To achieve analyte-specific detection, the sensor surface must be functionalized with biorecognition elements such as antibodies, aptamers, or receptor molecules. These elements enable selective adsorption of target analytes within the evanescent field region, thereby converting molecular recognition into measurable dielectric perturbations.

#### 4.2.1 Early cancer identification.

**Fig 12:**
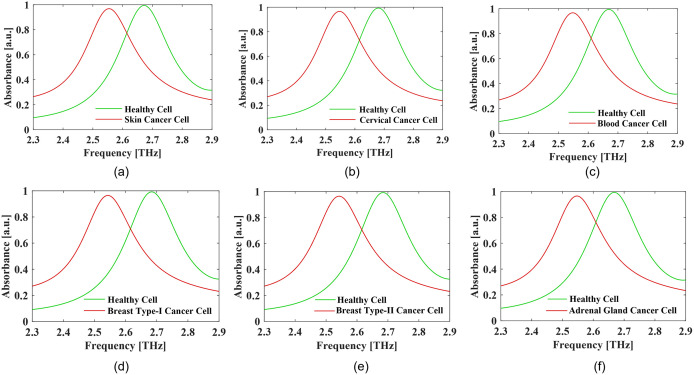
Absorbance spectra of the proposed metamaterial absorber for cancer cell identification. **(a–f)** Differentiate healthy and cancerous cells across multiple types, including skin, cervix, blood, breast (Type-I and Type-II), and adrenal gland. Noticeable resonance shifts reveal refractive index variations, allowing clear distinction between healthy and cancerous cells.

The absorption spectra in Fig 12 alongside refractive index data (Table 6) provide compelling evidence that THz metamaterial absorbers possess the capability to reliably distinguish between healthy and cancerous cells across diverse tissue types. In all cases, cancer cells exhibit higher refractive indices than their healthy counterparts, leading to a noticeable redshift in the resonance frequency, e.g., 2.672 THz for healthy skin (RI = 1.36), yielding a sensitivity of 5900 GHz/RIU. This trend holds across cervical (sensitivity: 5541.667 GHz/RIU), blood (8642.857 GHz/RIU), adrenal (8785.714 GHz/RIU), breast I (10071.43 GHz/RIU), and breast II (10142.86 GHz/RIU) tissues. These shifts are due to cancer-induced RI elevation, driven by cellular changes such as increased water content, altered membrane structure, and dense molecular packing, which elevate dielectric permittivity. Enhanced light–matter interaction modifies the resonant circuit capacitance, lowering resonance frequency. The metamaterial sensor demonstrates high GHz/RIU sensitivity to refractive index shifts, validated by absorbance peak changes, enabling non-invasive early cancer detection (e.g., ~ 10,000 GHz/RIU for breast cancer). This behavior confirms its potential as a spectral fingerprinting platform for tissue pathology [29].

**Table 6:** Sensing cancer cell.

*Cancer cell type*	*Refractive index (RI)* [15]		*Resonant frequence (THz)*		*Sensitivity* (GHz/RIU)
	*Cancer Cell*	*Healthy Cell*	*Cancer Cell*	*Healthy Cell*	
Skin	1.38	1.36	2.554	2.672	5900
Cervical	1.392	1.368	2.547	2.68	5541.667
Blood	1.39	1.376	2.548	2.669	8642.857
Adrenal Gland	1.395	1.381	2.545	2.668	8785.714
Breast I	1.399	1.385	2.543	2.684	10071.43
Breast II	1.401	1.387	2.542	2.684	10142.86

#### 4.2.2 Diabetes monitoring.

The THz metamaterial absorber enables precise blood glucose monitoring by detecting resonance frequency shifts correlated with refractive index (RI) variations. As glucose concentration (C%) increases (0–25%), the empirical relation:


$$\begin{array}{c} n=(\text{0}.\text{2}\text{0}\text{1}\text{5}\;\times \;C\%)\;+\;\text{1}.\text{3}\text{2}\text{9}\text{2} \end{array}$$

**Fig 13:**
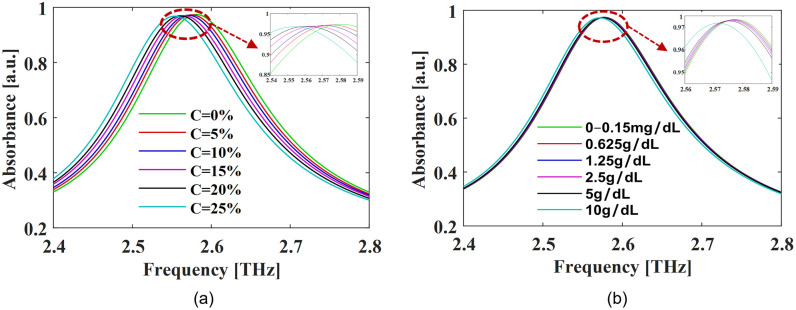
Absorbance spectra for varying blood and urine glucose concentrations. **(a)** Varying blood glucose concentrations and **(b)** varying urine glucose levels, ranging from 0 to 10 g/dL.

Where n denotes the refractive index and C% represents the glucose concentration percentage, as reported in [30]. This Fig 13(a) inducing metamaterial absorbance spectra in a red-shift that tabulated in Table 7. In this table, ‘Ref.’ designates the baseline sample against which all frequency shifts (∆f) and relative sensitivities are measured. This RI-frequency dependency is pivotal for sensing, as it directly translates glucose levels into measurable spectral changes without biochemical labels. Table 8 data confirms decreasing resonant frequencies (2.579–2.552 THz) highlighting minimal signal variance. The sensor’s RI sensitivity (0.001 RIU) facilitates detection of physiological glucose levels, with urine analyte tests in Fig 13(b) further validating performance. The linear, repeatable response ensures high precision (0.001 RIU sensitivity), critical for detecting sub-millimolar glucose variations in physiological ranges. Stable absorbance (~0.97) minimizes noise, enhancing reliability for non-invasive, real-time monitoring. Such specificity arises from direct coupling between glucose concentration and resonant electromagnetic modes, circumventing matrix effects common in complex biofluids. By bypassing exogenous labels or sample pretreatment, the platform enables robust, multiplexed diagnostics across diverse physiological media (blood/urine).

**Table 7:** Sensing blood glucose concentration percentage.

*Blood Glucose (C%)*	*RI [31]* [29]	*f*_*r*_ *(THz)*	*Maximum Absorbance*	*Δf (GHz)*	*Sensitivity (GHz/RIU)*
0%	1.3292	2.581	0.9742	Ref.	Ref.
5%	1.3392	2.555	0.9728	10	1000.00
10%	1.3493	2.571	0.9714	17	845.77
15%	1.3594	2.566	0.9697	23	761.59
20%	1.3695	2.561	0.9793	32	794.04
25%	1.3795	2.554	0.9681	47	934.39

*Note: “Ref.” indicates the Reference Baseline used for calculating frequency shifts (*$\Delta f=\left|f_{sample}-f_{reference}\right|$*) and sensitivity* $\left(S=\frac{\Delta f}{\Delta n}\right)$.

**Table 8:** Computation of urine glucose levels performance for the proposed sensor.

*Urine glucose levels*	*RI* [13]	*f*_*r*_ *(THz)*	*Maximum Absorbance*	*Δf (GHz)*	*Sensitivity (GHz/RIU)*
(0–15) mg/dL	1.335	2.579	0.9734	Ref.	Ref.
0.625 g/dL	1.336	2.588	0.9733	9	9000.00
1.25 g/dL	1.337	2.577	0.9731	2	1000.00
2.5 g/dL	1.338	2.566	0.973	13	4333.33
5 g/dL	1.341	2.558	0.9727	21	3500.00
10 g/dL	1.347	2.552	0.9718	27	2250.00

*Note: “Ref.” indicates the Reference Baseline used for calculating frequency shifts (*$\Delta f=\left|f_{sample}-f_{reference}\right|$*) and sensitivity* $\left(S=\frac{\Delta f}{\Delta n}\right)$.

#### 4.2.3 Blood component and biomolecule detection.

Blood comprises multiple components with distinct refractive indices, as detailed in Table 9, which can be exploited for precise analysis and detection. For example, red blood cells have a greater refractive index than the surrounding fluid. The refractive index (RI) of blood varies with hemoglobin (Hb) concentration, and their relationship is described by the empirical equation:

**Table 9:** Sensing blood component.

*Blood component*	*RI* [32]	*f*_*r*_ *(THz)*	*Maximum Absorbance*	*Δf (GHz)*	*Sensitivity (GHz/RIU)*
Water	1.33	2.58	0.9741	Ref.	Ref.
Plasma	1.35	2.571	0.9713	9	450.00
White blood cells	1.36	2.565	0.9696	15	500.00
Hemoglobin	1.38	2.554	0.9682	26	520.00
Red blood cells	1.4	2.543	0.9647	37	528.57

*Note: “Ref.” indicates the Reference Baseline used for calculating frequency shifts (*$\Delta f=\left|f_{sample}-f_{reference}\right|$*) and sensitivity* $\left(S=\frac{\Delta f}{\Delta n}\right)$.


**Fig 14:**
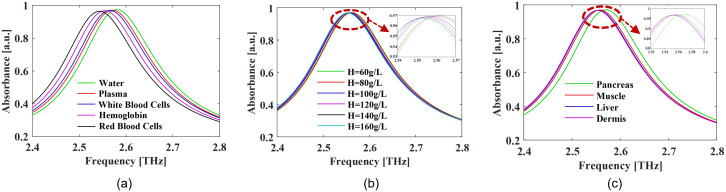
Analysis of the THz absorbance spectra for blood components, hemoglobin concentrations, and tissues. **(a)** Blood components (water, plasma, WBCs, hemoglobin, RBCs) displaying unique spectral features, **(b)** variations in hemoglobin concentration, and (c) different tissues (pancreas, muscle, liver, dermis) highlighting their characteristic absorbance profiles.

$$\begin{array}{c} n=\text{1}.\text{3}\text{8}+\frac{\left(\text{H }-\;{\text{H}}_{\text{normal}}\right)}{\text{5766}.\text{5}\;} \end{array}$$

where H is the hemoglobin concentration (g/L), and H_normal_ represents the typical human hemoglobin level, taken as 140 g/L, corresponding to a baseline RI of 1.38 [31]. The THz metamaterial absorber demonstrates distinct spectral fingerprints for blood components in Fig 14(a), with hemoglobin and red blood cells exhibiting pronounced absorbance peaks (~2.5–2.6 THz), while plasma and water show lower absorption, enabling differentiation based on dielectric properties. The data presented in Table 10 used to detect anemia, polycythemia, and blood disorders. Variations in the molecular composition and structure of biological tissues result in differing refractive indices.

**Table 10:** Sensing hemoglobin concentration.

*Hemoglobin Concentration*	*RI [31]* [32]	*f*_*r*_ *(THz)*	*Maximum Absorbance*	*Δf (GHz)*	*Sensitivity (GHz/RIU)*
60g/L	1.3366	1.645	0.9735	Ref.	Ref.
80g/L	1.3695	1.629	0.973	16	486.32
100g/L	1.373	1.614	0.9729	31	851.65
120g/L	1.3765	1.6	0.9723	45	1127.82
140g/L	1.38	1.586	0.9725	59	1359.45
160g/L	1.3834	1.487	0.9719	158	3376.07

*Note: “Ref.” indicates the Reference Baseline used for calculating frequency shifts (*$\Delta f=\left|f_{sample}-f_{reference}\right|$*) and sensitivity* $\left(S=\frac{\Delta f}{\Delta n}\right)$.

Fig 14(b) shows that hemoglobin concentration variations induce resonance frequency shifts, demonstrating potential for hematological diagnostics. Table 10 categorizes anemia severity based on the extent to which hemoglobin (Hb) concentration falls below the normal threshold. Conversely, elevated Hb levels may indicate polycythemia, a condition characterized by excessive RBC production, increasing the risk of thrombosis and cardiovascular complications. In Fig 14(b), resonance redshifts correlate with increasing hemoglobin concentrations (H = 60–160g/L), reflecting refractive index modulation by glucose. The linear shift (~0.02 THz per 20g/L hemoglobin increment) underscores high sensitivity to physiological hemoglobin levels (60–160g/L), critical for blood disorder monitoring.

By comparing the measured refractive index of an unknown tissue sample with reference values listed in Table 11, tissue identification can be achieved with high absorbances. To ensure measurement reliability and consistency, standardized protocols for tissue preparation and refractive index assessment are essential. Tissue-specific spectra in Fig 14(c) reveal unique resonant modes for pancreas (sharp peak at ~2.5 THz) and liver (broadband absorption at ~2.7 THz), attributable to variations in biomolecular composition (e.g., lipids, proteins). Minimal spectral overlap between analytes (e.g., hemoglobin vs. tissues) ensures high selectivity, while stable absorbance baselines indicate robustness against matrix effects. This platform’s ability to differentiate complex biofluids and tissue signatures, alongside glucose quantification via resonance shifts, establishes it as a versatile tool for non-invasive, multi-analyte clinical diagnostics.

**Table 11:** Sensing tissues.

*Tissues*	*RI [33]* [31]	*f*_*r*_ *(THz)*	*Maximum Absorbance*	*Δf (GHz)*	*Sensitivity (GHz/RIU)*
Pancreas	1.3517	2.57	0.9711	Ref.	Ref.
Muscle	1.3713	2.558	0.9697	12	612.24
Liver	1.3791	2.554	0.9683	16	583.94
Dermis	1.3818	2.553	0.9673	17	564.78

*Note: “Ref.” indicates the Reference Baseline used for calculating frequency shifts (*$\Delta f=\left|f_{sample}-f_{reference}\right|$*) and sensitivity* $\left(S=\frac{\Delta f}{\Delta n}\right)$.

#### 4.2.4 Intracellular and brain component detection.

**Fig 15:**
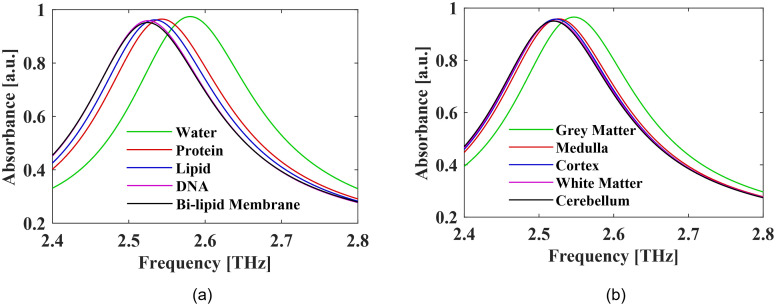
Absorbance spectra analysis for intracellular components and different brain tissues. (a) Intracellular components (water, protein, lipid, DNA, and bilipid membranes), (b) brain tissues (grey matter, medulla, cortex, white matter, and cerebellum).

To detect intracellular components using RI sensor Fig 15(a), we need to prepare the sample appropriately. Homogenization releases intracellular components, and filtering removes large debris and cell membranes. The intracellular components are introduced into the RI sensor. When optical excitation is given, a shift of resonant frequency is observed depending on its refractive index [34]. The refractive index of the sample can be determined from the shift of resonant frequency which can be seen in Table 12, and the maximum absorbances. The absorption spectroscopy provides distinct resonant signatures for intracellular and brain components, facilitating differentiation. Among intracellular materials, water exhibits the highest resonant frequency (2.5800 THz) and maximum absorbance (0.9741), whereas the bi-lipid membrane has the lowest (2.5250 THz, 0.9518). Intermediate intracellular components (protein, lipid, DNA) lie between these extremes.

**Table 12:** Sensing intracellular component.

*Intracellular Component*	*RI [35]* [36]	*f*_*r*_ *(THz)*	*Maximum Absorbance*	*Δf (GHz)*	*Sensitivity (GHz/RIU)*
Water	1.33	2.58	0.9741	Ref.	Ref.
Protein	1.4	2.543	0.9647	37	528.57
Lipid	1.42	2.534	0.9618	46	511.11
DNA	1.44	2.53	0.9587	50	454.55
Bi-lipid Membrane	1.46	2.525	0.9518	55	423.08

*Note: “Ref.” indicates the Reference Baseline used for calculating frequency shifts (*$\Delta f=\left|f_{sample}-f_{reference}\right|$*) and sensitivity* $\left(S=\frac{\Delta f}{\Delta n}\right)$.

In brain tissues (Fig 15b), grey matter shows the highest resonant frequency (2.5470 THz) and absorbance (0.9654), while the cerebellum shows the lowest (2.5200 THz, 0.9501). Medulla, cortex, and white matter lie between these extremes. These trends align with refractive indices (ranging approximately 1.33–1.46 intracellularly and 1.395–1.470 in brain), Tables 12 and 13 highlighting unique spectral fingerprints that enable accurate identification of each tissue type.

**Table 13:** Sensing brain component.

*Brain* *Component*	*RI [37]* [33]	*f*_*r*_ *(THz)*	*Maximum Absorbance*	*Δf (GHz)*	*Sensitivity (GHz/RIU)*
Grey Matter	1.395	2.547	0.9654	Ref.	Ref.
Medulla	1.438	2.526	0.9586	21	488.37
Cortex	1.444	2.523	0.9576	24	489.80
White Matter	1.467	2.521	0.9509	26	361.11
Cerebellum	1.47	2.52	0.9501	27	360.00

*Note: “Ref.” indicates the Reference Baseline used for calculating frequency shifts (*$\Delta f=\left|f_{sample}-f_{reference}\right|$*) and sensitivity* $\left(S=\frac{\Delta f}{\Delta n}\right)$.

#### 4.2.5 Identification of malaria germs.

**Fig 16:**
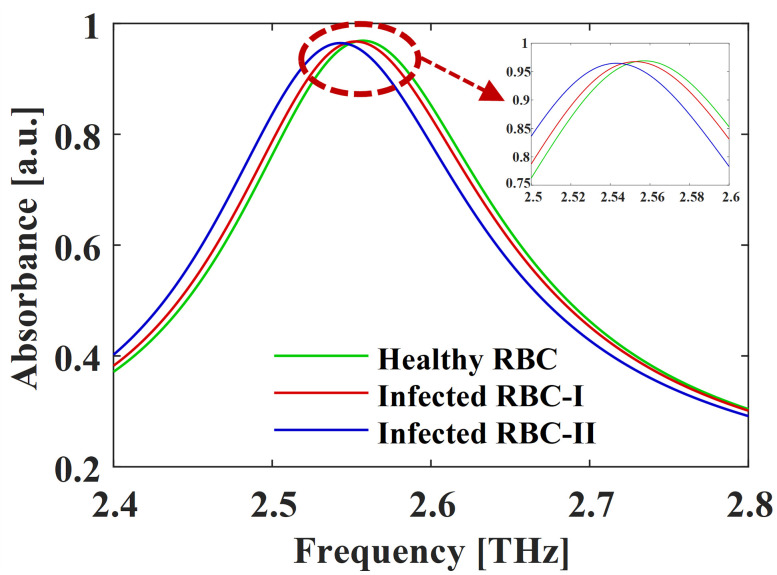
Absorbance spectra comparison between healthy red blood cells (RBC) and malaria-infected RBCs (RBC-I, II).

Absorbance spectroscopy in Fig 16 demonstrates clear spectral distinctions between healthy and malaria-infected red blood cells (RBCs), enabling precise detection of disease progression. Where healthy RBCs exhibit the highest resonant frequency at 2.5570 THz and a maximum absorbance of 96.90%. As infection advances, a progressive redshift is observed: Infected RBC-I shifts to 2.5530 THz (0.9674 absorbance), while Infected RBC-II further shifts to 2.5430 THz with a reduced absorbance of 0.9646. Correspondingly, the refractive index increases from 1.373 (healthy) to 1.383 (RBC-I) and 1.399 (RBC-II), indicating altered optical properties due to parasitic invasion. These spectral shifts provide a sensitive and non-invasive diagnostic indicator for differentiating between infection stages, underscoring the efficacy of terahertz-based sensing in malaria diagnostics. Corresponding absorbance values for infected RBCs at both stages are summarized in Table 14, demonstrating the sensor’s sensitivity and diagnostic capability. Additionally, recent advances in metamaterial biosensors indicate strong correlation between simulation and experimental results, validating their practical applicability [42].

**Table 14:** Sensing malaria detection.

*Malaria Detection*	*RI [14]* [34]	*f*_*r*_ *(THz)*	*Maximum Absorbance*	*Δf (GHz)*	*Sensitivity (GHz/RIU)*
Healthy RBC	1.373	2.557	0.969	Ref.	Ref.
Infected RBC-I	1.383	2.553	0.9674	4	400.00
Infected RBC-II	1.399	2.543	0.9646	14	538.46

*Note: “Ref.” indicates the Reference Baseline used for calculating frequency shifts (*$\Delta f=\left|f_{sample}-f_{reference}\right|$*) and sensitivity* $\left(S=\frac{\Delta f}{\Delta n}\right)$.

## 5. Comparative analysis

Table 15 demonstrates that previously reported THz metamaterial absorbers for biomedical sensing exhibit several important limitations despite achieving moderate to high sensing performance. Earlier studies such as [10] and [38] achieved dual-band absorption with sensitivities of 1150 GHz/RIU and 971 GHz/RIU, respectively; however, their relatively broad FWHM values (0.522–0.765 THz in [10]) indicate lower spectral selectivity and reduced detection precision for subtle biomarker variations. Reference [9] achieved extremely high absorbance (99.998%) and narrow FWHM (0.012 THz), but the design operated only in a single narrow resonance band with no polarization stability analysis, limiting its practical applicability for multifunctional biomedical sensing. Similarly, [12,19], and [40] showed comparatively low sensitivities of 374 GHz/RIU, 5002 GHz/RIU, and 300 GHz/RIU, respectively, which restrict their capability for ultra-trace biomarker detection and early-stage disease diagnosis. Although [17] and [20] demonstrated improved sensitivity values of 2370 GHz/RIU and 2075 GHz/RIU, their absorber structures suffered from limited operating bandwidth and insufficient multi-analyte detection capability. Moreover, several earlier works relied on costly or fabrication-sensitive substrates such as polyimide, GaAs, or metallic nanostructures, which may increase fabrication complexity and reduce scalability for real biomedical deployment. Reference [22] reported polarization-insensitive performance with 1080 GHz/RIU sensitivity, yet lacked multi-band ultra-sharp resonance behavior and did not address complex biomarker environments. Likewise, [23] focused mainly on EMI shielding and radar applications rather than biomedical biosensing, making it less suitable for precise biomarker detection. In general, the literature reveals persistent challenges in THz metamaterial biosensors, including narrow operational bandwidth, insufficient sensitivity, polarization dependence, broader resonance linewidth, fabrication complexity, and limited capability for simultaneous multi-biomarker detection. These challenges are commonly recognized in THz metamaterial sensing systems, particularly for highly absorptive biological environments and ultra-low concentration analytes.

**Table 15:** Comparison of the proposed metamaterial sensor with recent related works.

Reference	Operation Range (THz)	Number of Bands	Material Substrate	Q-Factor	FWHM	Absorbance (%)	Polarization Angle	Sensitivity (GHz/RIU)	Applications (Sensing/Detection)
[9]	1.354	1	SiO_2_	112.5	0.012	99.998	—	1700	Malaria, dengue, herpes simplex virus, influenza, HIV, and coronaviruses
[10]	3–9	2	SiO_2_	8.243, 9.601	0.522, 0.765	98.32, 98.49	0-90	1150	Cancer detection, blood glucose monitoring, and detecting malaria mosquito bite
[12]	1.5–1.9	1	Polyimide	28.26	0.0591	96	0-90	374	Tissues, monitoring drug, early-stage disease
[17]	1.0–3.0	2	InSb	574.46	0.142, 0.047	≈100	—	2370	Tuberculosis Bacteria
[19]	1.0–3.0	1	Polyimide	26.2	0.078	96.9	—	5002	Cancer, Malaria, Bacillus Virus, and Tuberculosis Diagnosis
[20]	≈0–1.4	2	FR-4 (glass-reinforced epoxy)	24.35	0.030, 0.041	≈98	—	2075	Glucose, HIV-1, and M13 viruses
[38]	≈1.90–1.98	2	Aluminium	240.15, 181.61	0.007, 0.010	98.17, 99.05	0-90	971	Biological Samples
[37] [39]	≈0.5–1.9	3	Copper & Polytetra fluoroethylene (PTEE)	26.01 17.83, 58.04	0.2124, 0.7005, 0.5804	97.43 79.22, 99.02	0-60	473.86	Liquid
[40]	2.3–2.7	1	GaAs	61.75	0.040	99.50	0-90	300	Skin Cancer Detection
[22]	6–8	3	PTFE (Teflon)	494.3	—	99.75–99.87	Polarization-insensitive	1080	Cervical cancer (HeLa cells) detection
[23]	0.008–0.03	4	FR-4	—	—	95.7–99.8	Polarization-insensitive	—	EMI shielding, sensing, and radar
This work	0.85–2.8	3	FR-4	11.8589, 12.6666, 12.4662	0.078, 0.135, 0.222	99.925, 99.9687, 99.5763	0-90	10,142.86	Virus, Cancer, Diabetes, Biomolecule & Blood component, Intracellular & Brain component, Malaria

In contrast, the proposed work significantly outperforms existing designs by combining triple-band operation (0.85–2.8 THz), ultra-high absorbance (99.925%, 99.9687%, and 99.5763%), narrow resonance linewidths, and exceptionally high sensitivities reaching 10,142.86 GHz/RIU. The proposed absorber also maintains polarization stability over a wide angular range (0°–90°), which is highly desirable for reliable real-time biomedical sensing applications. Unlike previous studies that focused on limited analyte categories, the proposed THz meta-absorber demonstrates versatile applicability in detecting viruses, cancer cells, diabetes biomarkers, blood components, intracellular and brain components, and malaria-related biomarkers simultaneously. The use of a low-cost FR-4 substrate further enhances fabrication simplicity and practical implementation potential compared to expensive semiconductor-based substrates. Therefore, the proposed design provides a substantial advancement toward highly sensitive, label-free, multi-analyte THz biomarker detection systems suitable for next-generation biomedical diagnostics and early disease screening applications.

## 6. Future scope

In the next research phase, metasurface will be fabricated via nanoimprint lithography or electron-beam lithography to achieve high-resolution absorber patterns. Design optimization will be guided by simulations to ensure targeted resonance for sensing applications, informing material and structural choices. A biologically functional interface layer will be applied to enhance biomolecule adsorption and detection sensitivity. Although the present work demonstrates promising sensing characteristics through numerical proof-of-concept based full-wave electromagnetic analysis, the proposed absorber currently represents a theoretical proof-of-concept platform. Future work will focus on experimental fabrication with standard microfabrication techniques, terahertz characterization, microfluidic integration, and validation using real biological samples to assess practical clinical applicability. Additionally the effects of environmental impacts (humidity, temperature) on sensor stability and operational stability will be investigated to improve reliability through suitable protective and calibration strategies. In practice, humidity may affect the dielectric properties of the substrate or cause surface adsorption. If necessary, this could be mitigated with hydrophobic coatings. Since the simulations used fixed dielectric constants, humidity-induced shifts are not reflected, but based on prior studies, any resonance shifts due to moisture uptake are generally small and reversible. Thermal effects could slightly alter material permittivity and physical dimensions (via thermal expansion). While not explicitly simulated, materials used in our design exhibit relatively low thermal sensitivity in the THz regime. Future work could extend this study by including temperature-dependent permittivity and expansion models to quantify such effects. While direct environmental and operational stability testing was outside the scope of this simulation-based work, the use of stable materials and reference to prior studies support the expectation that the proposed THz metamaterial absorber would maintain reliable performance under typical operating and environmental conditions.

## 7. Conclusion

This comprehensive analysis of terahertz metamaterial sensors reveals significant advances in biomedical diagnostic technology, with profound implications for global healthcare delivery. Metamaterial engineering has produced a triple-band sensor system that achieves >99.5% absorption efficiency across three distinct resonant frequencies at 0.925 THz, 1.71 THz, and 2.768 THz, representing a substantial technological advancement over existing sensor platforms. The sensor exhibits increasing sensitivity across three bands, with a maximum sensitivity of 10,142.86 GHz/RIU at third band for Breast II type anomalies tissue detection. The research demonstrates remarkable performance improvements, including absorptivity in spectral bandwidth, while simultaneously achieving 74% reduction in computational overhead compared to traditional design methodologies. These advances address critical bottlenecks in metamaterial sensor development, particularly the computational intensity and fabrication challenges that have historically limited practical implementation of multiband terahertz sensors. The sensor’s demonstrated capabilities across diverse biomedical applications, from glucose detection to cancer cell identification and infectious disease diagnosis, position it as a transformative platform for personalized medicine and point-of-care testing. In addition to demonstrating high refractive index sensitivity and frequency-resolving capability across the three resonance bands, the proposed sensor highlights the potential advantages of multi-band terahertz biosensing for analyte discrimination. However, practical clinical selectivity would require the incorporation of specific bioreceptors and suitable surface functionalization chemistry, which remains part of ongoing and future experimental development. The polarization-insensitive design and enhanced fabrication tolerance achieved through design optimization significantly improve the practical viability of these sensors for clinical deployment. This computational advancement in photonics and metamaterial engineering, enabling next-generation biosensing platforms with multi resonance absorption exceeding 99%. Technology offers transformative potential for non-invasive, real-time diagnostics in early detection of malignancies, infectious diseases, and metabolic disorders, thereby enhancing patient outcomes and global health management.
